# Rapid Recovery of Fat Mass in Small for Gestational Age Preterm Infants after Term

**DOI:** 10.1371/journal.pone.0014489

**Published:** 2011-01-05

**Authors:** Paola Roggero, Maria L. Giannì, Nadia Liotto, Francesca Taroni, Anna Orsi, Orsola Amato, Laura Morlacchi, Pasqua Piemontese, Massimo Agosti, Fabio Mosca

**Affiliations:** 1 Neonatal Intensive Care Unit (NICU), Department of Mother and Infant Science, Fondazione IRCCS “Ca' Granda” Ospedale Maggiore Policlinico, University of Milan, Milan, Italy; 2 Maternal and Child Health Department, Del Ponte Hospital, A.O. Di Circolo Fondazione Macchi, Varese, Italy; UCL Institute of Child Health, United Kingdom

## Abstract

**Background:**

Preterm small for gestational age (SGA) infants may be at risk for increased adiposity, especially when experiencing rapid postnatal weight gain. Data on the dynamic features of body weight and fat mass (FM) gain that occurs early in life is scarce. We investigated the postnatal weight and FM gain during the first five months after term in a cohort of preterm infants.

**Methodology/Principal Findings:**

Changes in growth parameters and FM were prospectively monitored in 195 infants with birth weight ≤1500 g. The infants were categorized as born adequate for gestational age (AGA) without growth retardation at term (GR−), born AGA with growth retardation at term (GR+), born SGA. Weight and FM were assessed by an air displacement plethysmography system. At five months, weight z-score was comparable between the AGA (GR+) and the AGA (GR−), whereas the SGA showed a significantly lower weight.The mean weight (g) differences (95% CI) between SGA and AGA (GR−) and between SGA and AGA (GR+) infants at 5 months were −613 (−1215; −12) and −573 (−1227; −79), respectively. At term, the AGA (GR+) and the SGA groups showed a significantly lower FM than the AGA (GR−) group. In the first three months, change in FM was comparable between the AGA (GR+) and the SGA groups and significantly higher than that of the AGA (GR−) group.The mean difference (95% CI) in FM change between SGA and AGA (GR−) and between AGA (GR+) and AGA (GR−) from term to 3 months were 38.6 (12; 65); and 37.7 (10; 65). At three months, the FM was similar in all groups.

**Conclusions:**

Our data suggests that fetal growth pattern influences the potential to rapidly correct anthropometry whereas the restoration of fat stores takes place irrespective of birth weight. The metabolic consequences of these findings need to be elucidated.

## Introduction

Evidence indicates that early nutrition, growth and subsequent health are crucially related. Several studies have demonstrated that early life growth patterns exert programming effects on disease risk in later life, highlighting the key role played by early nutrition [1], [2]. There is still debate as to when the sensitive periods of early development occur, during which the “programming” takes place [3] and the relative contribution of intrauterine and postnatal growth to subsequent health outcomes needs further clarification.

Body composition, in terms of fat mass (FM), may contribute to this “programming” process [4].

Small size at birth as well as rapid catch-up growth during infancy has been associated with an increased risk for developing the metabolic syndrome in adulthood [5], [6]. It has been recently suggested that relative adiposity, which is a well known risk factor for cardiovascular disease [7], may develop due to under-nutrition as well as growth retardation [8]. Preterm infants are at increased risk for developing insulin resistance due to the stressful conditions and the cumulative nutritional deficits they experience during early postnatal life. As a consequence, hyperinsulinaemia and a down-regulation of visceral β3-adrenoreceptors may lead to increased intra-abdominal adiposity [8].

Preterm small for gestational age (SGA) infants assessed at term corrected age have been reported to be at risk for developing increased adiposity [9]. In addition, abnormal body composition and altered insulin sensitivity have been found in SGA infants who experienced rapid postnatal weight gain [10], [11], [12].

Data on the dynamic features of body weight gain and FM gain that occur during the first months of life in SGA preterm infants is scarce. Therefore, the aim of the present study was to investigate the postnatal weight gain and FM accretion during the first five months of corrected age in a cohort of preterm infants who were categorized according to intrauterine growth pattern and according to postnatal growth.

## Materials and Methods

### Ethics statements

The study was approved by the Departmental Ethics Committee, Fondazione IRCCS “Ca' Granda” Ospedale Maggiore Policlinico, and written consent was obtained from both parents.

### Patients

Two hundred and seven preterm infants among all consecutive newborns admitted to the same Institution from January 2007 to June 2009 were enrolled in the study. Inclusion criteria were: birth weight <1500 g, singleton pregnancy. Exclusion criteria were: presence of congenital diseases, chromosomal abnormalities, chronic lung disease (defined by the use of supplemental oxygen at 36 weeks' postconceptional age), severe brain, metabolic, cardiac or gastrointestinal diseases (i.e. necrotizing enterocolitis classified as stage 3 according to the classification of Bell et al. [13] ), being breastfed after term. The reason for choosing relatively strict eligibility criteria relied on the fact that we wanted to investigate the growth and body composition in subgroups of preterm infants not affected by illnesses that could interfere with the variables investigated.

### Design

We conducted a prospective, observational study. Basic subject characteristics (birth weight, length, head circumference, gestational age, gender, being adequate [AGA] or small for gestational age [SGA]) were recorded. Anthropometric parameters (weight, length and head circumference) and fat mass were assessed at term and at 1, 3 and 5 months of corrected age. Gestational age was based on the last menstrual period and first trimester ultrasonogram. Corrected age was calculated using the chronologic age and adjusting for gestational age, that is, for the number of additional weeks from term (40 weeks). Infants with birth weight ≥ or <10^th^ percentile for gestational age, according to the Fenton's chart [14], were classified as AGA or SGA, respectively.

### Growth and fat mass measurements

Body weight, length and head circumference were measured according to standard procedures [15]. Subject mass was measured on an electronic scale accurate to the nearest 0.1 g and body length was measured to the nearest 1 mm on a Harpenden neonatometer (Holtain Ltd, UK). Head circumference was measured to the nearest 1 mm with non stretch measuring tape. Growth z-scores were calculated by EuroGrowth 2000 software (Euro-Growth Study Group, Vienna, Austria). Infants with weight ≥or <2 SD at term were classified as infants being non-growth retarded (GR−) or growth retarded (GR+), respectively. The change in weight [100 × (weight at second examination – weight at first examination)/weight at first examination)] between birth and term, between term and 3 months of corrected age and between 3 and 5 months of corrected age were then calculated. FM was assessed using an air displacement plethysmography system (PEA POD Infant Body Composition System, LMI, Concord, CA, USA). A detailed description of the PEA POD's physical design, operating principles, validation and measurement procedures is provided elsewhere [16], [17]. The PEA POD assesses FM and fat free mass by direct measurements of body mass and volume and the application of classic densitometric principles. Infants were measured in the PEA POD naked. Each PEA POD test took about 3 min to complete. Subject volume was measured in an enclosed chamber by applying gas laws that relate pressure changes to volumes of air in the chamber. Body density was then computed from the measured body mass and volume, and inserted into a standard formula for estimating the percentage of total body FM according to a 2-compartment model. The intra-observer coefficient of variation for the percentage of FM estimates was 0.3%. The change in FM [100 × (FM at second examination−FM at first examination)/FM at first examination)] between term and 3 months of corrected age and between 3 and 5 months of corrected age were then calculated.

### Nutritional Practices

Preterm infants received parenteral and minimal enteral feeding, with expressed breast milk or preterm formula, for a minimum of two weeks. Subsequently, the nutritional regimen up to discharge was either fortified breast milk (2.2 g/100 ml and 82 Kcal/100ml) or preterm formula (2.4 g/100 ml and 80 Kcal/100 ml) when breast milk was unavailable or insufficient. From term up to the fifth month, infants were fed a nutrient-enriched postdischarge formula (protein 2 g/100 ml; energy 75 kcal/100 ml) on demand and were given no other foods. At discharge, parents were instructed to record the daily quantities of milk consumed by the infants in a diary. The average daily energy and protein intakes were then calculated.

### Statistical analysis

Descriptive data are expressed as mean (SD) or number of observations (percentage). Differences among infants in repeated measurements of growth parameters and FM were assessed by an analysis of variance. Significance of multiple comparisons was adjusted by the Bonferroni correction. A χ2 test was used for comparisons between discrete variables. For analysis, infants were categorized as born AGA without growth retardation at term (GR−), born AGA with growth retardation at term (GR+), born SGA.

Statistical significance was set at α = 0.05 level. All statistical analyses were performed using SPSS (SPSS, version 12, SPSS Inc., Chicago, IL).

## Results

Growth and body composition were assessed in 195 (96 males) infants. There was no infant mortality throughout the follow-up. Out of the 207 infants originally recruited, 4 moved away or returned to their country of origin; 8 failed to attend the scheduled appointments. Mean gestational age (weeks) and birth weight (g) were 30.2 (2.3) and 1190 (284). Basic subject characteristics are shown in table 1. Birth weight was significantly lower in the infants born SGA when compared to the infants born AGA (GR−) and (GR+) whereas gestational age was significantly higher in infants born SGA.

**Table 1:** Basic subject characteristics according to categorization.

	AGA (GR−) (n = 53)	AGA (GR+) (n = 64)	SGA (n = 78)	P Values
Birth weight (g)	1260.8 (198)	1204.8 (253)	1131.1 (286)°	0.016
Gestational age (wks)	29.3(1.8)	29.4 (2.2)	31.4 (2.2)*	<0.001
Birth length (cm)	37.1 (4.5)	36.5 (3.9)	35.4 (4.8)	0.22
Birth HC^1^ (cm)	28.3 (2.6)	27.5 (2.9)	27.3 (2.4)	0.14
Males (n)	30 (56.6)	33 (51.6)	33 (42.3)	0.10

Data are presented as mean (SD) or number of observations (%).

HC^1^ = head circumference.

*SGA vs AGA (GR−) and AGA (GR+).

°SGA vs AGA (GR−).

### Growth

**Figure 1:**
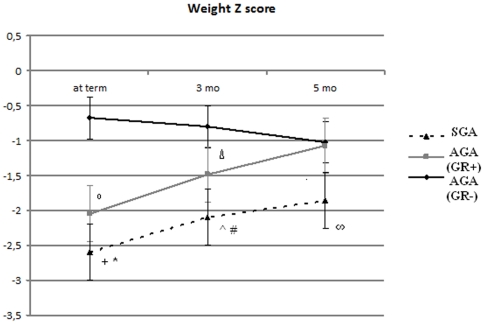
Mean weight z-scores at term, 3 and 5 months of corrected age according to group categorization. ^+^ P = 0.001 SGA vs AGA (GR+). * P<0.001 SGA vs AGA (GR−). ° P<0.001 AGA (GR+) vs AGA (GR−). ^#^ P<0.001 SGA vs AGA (GR−). ∧ P = 0.02 SGA vs AGA (GR+). ^Δ^ P = 0.01 AGA (GR+) vs AGA (GR−). ^∞^P = 0.03 SGA vs AGA (GR−) and AGA (GR+).

At term, and at 3 and 5 months of corrected age, the mean z-score for weight was significantly lower in SGA infants when compared to AGA (GR−) and AGA (GR+) infants (figure 1). The mean weight (g) differences (95% Confidence Interval) between SGA and AGA (GR−) infants at term, 3 and 5 months of corrected age were −837 (−999;−674), −792 (−1125;−459), −613 (−1215; −12), respectively. The mean weight (g) differences (95% Confidence Interval) between SGA and AGA (GR+) infants at term, 3 and 5 months of corrected age were −237 (−392;−83), −368 (−678;−60), −573 (−1227; −79), respectively. The AGA (GR+) infants showed the mean z-score for weight significantly lower at term and at 3 months of corrected age as compared to the AGA (GR−) infants whereas no difference was found at 5 months (figure 1). The mean weight (g) differences (95% Confidence Interval) between AGA (GR+) and AGA (GR−) infants at term and 3 months of corrected age were −599 (−769;−429), −423 (−768;−78), respectively.

The mean z-score for length in SGA infants was significantly lower from term to the third month when compared to AGA (GR+) and (GR−) infants (table 2). The mean length (cm) differences (95% Confidence Interval) between SGA and AGA (GR−) infants at term and 3 months of corrected age were −3.6 (−4.5;−2.7), −2.1 (−3;−1), respectively. The mean length (cm) differences (95% Confidence Interval) between SGA and AGA (GR+) infants at term and 3 months of corrected age were −1.7 (−2.6;−0.8), −1.6 (−2.7;−0.5), respectively. No difference in the mean z-score for length was found between groups at 5 months.

**Table 2:** Mean length and head circumference z-scores at each study visit time point according to categorization.

	Length z-scores			HC^1^ z-scores		
	Term	3 mo	5 mo	Term	3 mo	5 mo
AGA (GR−)	−1.25 (0,4)	−1.32 (0,35)	−1.34 (0,31)	0.24§ (0,5)	−0.5 (0,38)	−0.1 (0,42)
AGA (GR+)	−2.19° (0,3)	−1.61 (0,4)	−0.88 (0,42)	−1.0 (0,45)	−1.18 (0,39)	−0.52 (0,41)
SGA	−3.0* (0,42)	−2.3# ∧ (0,31)	−1.5 (0,35)	−1.07 (0, 53)	−2.28* (0,38)	−1.2# ∞ (0,36)

Data are presented as mean (SD).

HC^1^ = head circumference.

*P<0.001 SGA vs AGA (GR−) and AGA (GR+).

°P<0.001 AGA (GR+) vs AGA (GR−).

# P<0.001 SGA vs AGA (GR−).

∧ P = 0.003 SGA vs AGA (GR+).

§ P<0.001 AGA (GR−) vs SGA and AGA (GR+).

∞ P = 0.004 SGA vs AGA (GR+).

Mean z-score for head circumference was significantly lower at each study point in SGA infants when compared to AGA (GR+) and AGA (GR−) infants (table 2). The mean head circumference (cm) difference (95% Confidence Interval) between SGA and AGA (GR−) infants at term, 3 and 5 months of corrected age were −1.78 (−2.8;−0.7), −2.2 (−2.27; −0.4), −1.5 (−2.1; −0.7), respectively. The mean head circumference (cm) differences (95% Confidence Interval) between SGA and AGA (GR+) infants at 3 and 5 months of corrected age were −1.2 (−1.8;−0.3), −0.9 (−1.3; −0.5), respectively.

Mean z-score for head circumference was significantly lower in AGA (GR+) infants when compared to AGA (GR−) infants at term and at 3 months.. The mean head circumference (cm) differences (95% Confidence Interval) between AGA (GR+) and AGA (GR−) infants at term and at 3 months were −1.6 (−2.7;−0.5), −0.8 (−1.1; −0.2), respectively. However, there was no difference between the two groups at 5 months.

In Table 3 the mean changes in weight and FM between each study point according to group categorization are shown. The mean difference (95% Confidence Interval) in weight change between birth and term corrected age was significantly higher in AGA (GR−) infants when compared to AGA (GR+) [35.5 (7.6; 63)] and SGA [36 (9.5; 63)] infants.

**Table 3:** Change in weight and fat mass gain between each study point according to group categorization.

	%Δ Weight			% Δ FM	
	Birth-Term	Term-3 mo	3–5 mo	Term-3 mo	3–5 mo
AGA (GR−)	156.6* ∧ (47.4)	83.2+ (24)	17.0 (4.1)	29.1° # (27.7)	10.1 (20.1)
AGA (GR+)	121.1 (57.8)	107.8 (24.2)	17.4 (5.1)	66.8 (47.1)	8.5 (8.1)
SGA	120.3 (73.3)	111.2 (26.8)	21.4§ (4.7)	67.7 (59.4)	7.6 (19.2)

Data are presented as mean (SD).

*P = 0.007 AGA (GR−) vs AGA (GR+).

∧ P = 0.004 AGA (GR−) vs SGA.

+ P<0.001 AGA (GR−) vs AGA (GR+) and SGA.

§ P = 0.01 SGA vs AGA (GR−) and AGA (GR+).

°P = 0.003 AGA (GR−) vs AGA (GR+).

# P = 0.002 AGA (GR−) vs SGA.

On the contrary, the mean difference (95% Confidence Interval) in weight change between term and 3 months of corrected age was significantly lower in AGA (GR−) infants when compared to AGA (GR+) [−24.6 (−37; −12)] and SGA [−27.9 (−40; −15.6)] infants.

No difference between the AGA (GR−) and AGA (GR+) groups was detected between 3 and 5 months of corrected age whereas the SGA infants showed a significantly higher mean difference (95% Confidence Interval) in weight change than the AGA (GR−) [4.4 (0.8; 8)] and the AGA (GR+) infants [4.1 (0.2; 7.9)].

The mean change difference (95% Confidence Interval) in FM between term and 3 months of corrected age was significantly lower in AGA (GR−) infants when compared to AGA (GR+) [−37.8 (−65; −10)] and SGA infants [−38.6 (−65; −12)] (table 3), whereas no differences between groups were detected between 3 and 5 months.

**Figure 2:**
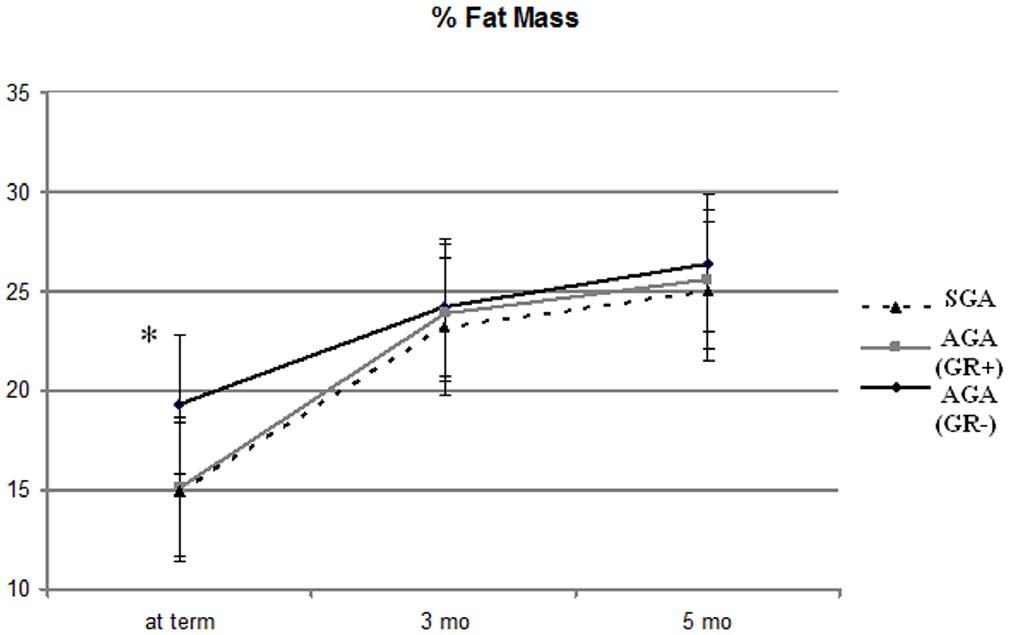
Mean % of FM at term, 3 and 5 months of corrected age according to group categorization. *P<0.001 AGA (GR−) vs AGA (GR+) and SGA.

Figure 2 shows the mean % FM at each study point according to categorization. At term % FM was significantly higher in AGA (GR+) infants when compared to AGA (GR−) and SGA infants, whereas no differences between groups were detected at 3 and 5 months.

No significant differences in energy and protein intakes between groups were found during the study period (table 4).

**Table 4:** Mean energy and protein intakes at each study point according to group categorization.

	Energy intake (kcal/kg/d)			Protein intake (g/kg/d)		
Time	AGA (GR−)	AGA (GR+)	SGA	AGA (GR−)	AGA (GR+)	SGA
3 months	105.3 (19)	110.4 (21)	114.1 (20)	2.5 (0.6)	2.46 (0.7)	2.7 (0.7)
5 months	88.7 (21)	83.2 (22)	95.1 (14)	1.8 (0.6)	1.8 (0.7)	2.1 (1.0)

Data are presented as mean (SD).

## Discussion

This study investigates longitudinally, the postnatal weight gain and weight gain composition during the first months of corrected age in a cohort of preterm infants who were classified according to their intrauterine growth pattern and according to their postnatal growth.

In the present study, SGA infants showed the lowest mean z-score for weight at term in comparison to AGA (GR+ ) and (GR−) infants. Both the impaired intrauterine growth and the cumulative postnatal nutritional deficit explains this finding. As a consequence, infants who were born SGA, although exhibiting an increased growth rate between term and the fifth month, attained mean z-score values for weight that were persistently lower than that attained by infants born AGA (GR+) and (GR−). These results are consistent with previous studies which reported that SGA preterm infants experience a severe extra uterine growth failure [18]–[21]. Bertino et al. [19] have recently reported that being born preterm and small for gestational age exert negative effects on growth assessed at term and at 24 months of corrected age. Jordan et al. [20] observed that at 36 months, SGA infants remained lighter, shorter and had smaller head circumference values than AGA infants even if the postnatal growth rate of SGA infants was higher than that of AGA infants. Moreover, Hack et al. [21] demonstrated that SGA very low birth weight males do not catch up in growth by 20 years of age. In contrast, infants born AGA (GR+), who also showed an increased growth rate between term and the fifth month, successfully achieved similar mean z-score values for weight within the fifth month of corrected age when compared to AGA (GR−) infants. In addition, AGA (GR+) infants also recovered in terms of length and head circumference within the third month of corrected age. These results confirm previous findings reported by our group [22] related to a smaller sample of (GR+) and (GR−) AGA preterm infants. In contrast with our findings, Latan et al. [23] reported postnatal growth retardation in a group of AGA very low birth weight infants, resulting in weight that was below the 10th percentile at two years of age. A possible explanation is that infants having medical complications (i.e. bronchopulmonary dysplasia, severe intraventricular hemorrhage) that could negatively affect postnatal growth after discharge, were enrolled in the latter study. On the contrary, only premature infants without medical complications, that could interfere with subsequent growth, were included in our study. Our results suggest that the potential to rapidly correct anthropometry observed in the AGA (GR+) infants when compare to AGA (GR−) infants may reflect the influence of fetal programming, implying that the trajectory of growth may not be permanently affected by the development of postnatal growth restriction. In the case of AGA (GR+) infants, the lack of impaired intrauterine growth may allow these infants to recover from their postnatal growth restriction. In contrast, the persistence of postnatal GR in the SGA infants may suggest that either these infants have an intrinsic lower growth potential or that the growth constraint experienced during the intrauterine life may delay the occurrence of recovery of growth. Although SGA infants achieved mean z scores for growth parameters above −2 z-scores within the fifth month, they did not attain values similar to that of AGA infants in terms of weight and head circumference.

With respect to body composition, both SGA and AGA (GR+) infants showed % FM at term significantly lower than that of AGA (GR−) infants, suggesting that the postnatal GR is accompanied by a relative lack of FM accretion. Nevertheless, the mean FM value presented at term by all the infants enrolled in the study, regardless of categorization, was much higher than that found in full term neonates at birth [24]. The finding of an increased adiposity in preterm infants assessed at term corrected age is consistent with previous reports [9], [25]. The increased amount of fat accretion has been linked to the energy intake [26] and could also be partially dependent on the several differences between fetal nutrition and postnatal nutrition [27]. The higher fat deposition could also represent an adaptive mechanism to postnatal life, for example, to augment body energy stores and ameliorate thermoregulation [28].

Surprisingly, from term to the third month of corrected age, both SGA and AGA (GR+) infants showed a higher change in FM than the AGA (GR−) infants, so that no difference in percentage of FM between groups was detected at three months. From the third month up to the end of the study, SGA, AGA (GR+) and (GR−) infants showed comparable change in FM. Moreover, the mean FM values attained at three and five months by all infants, regardless of group categorization, were comparable to those of full term breastfed infants [29].

To our knowledge there is a paucity of data related to body composition changes that occur over the first months of life in SGA infants. According to our results, Beltrand et al. [30] reported the restoration of body size and fat stores within the fourth month of age in fetal growth restricted full term infants without detrimental consequences at one year of age on body composition or metabolic profile. Ibanez et al. [31] reported that full term SGA children, who developed a spontaneous catch up growth, at 2 years of age, showed similar body composition when compared to full term AGA infants. However, the authors found a striking shift towards visceral adiposity in full term SGA children between 2 and 4 years of age and a further increase in central adiposity between 4 and 6 years [12]. On the contrary, Willemsen et al. [32] reported a decreased percentage of total body FM in former preterm, short SGA children assessed at 6.8 years of age in comparison to AGA children but a similar body fat distribution both in the SGA and AGA children, suggesting a trend for SGA children towards the development of central adiposity. Meas et al. [33] described a fast progression of adiposity from 22 up to 30 years of age in adults born full term SGA resulting in a higher percentage of total body FM than in subjects born full term AGA.

These findings indicate that being born SGA may affect body composition in different ways according to the period of development. In the present study, body composition in preterm infants was assessed in early infancy. Whereas the rapid recovery of FM exhibited by AGA (GR+) infants suggests that the absence of impaired intrauterine growth may allow these infants to recover from their postnatal lack of FM accretion, the accelerated gain in FM experienced by the SGA infants may partly be due to the fact that subjects who have been exposed to impaired fetal growth may be susceptible to gain more fat. The “FM catch up” may represent a compensatory event associated with the degree of impairment of fetal growth [34]. In addition, as energy intake has been advocated as a major determinant in FM accretion [26], the rapid advances in FM from term to the fifth month in the SGA infants could be explained by the cumulative effect on FM gain caused by the slightly higher energy intakes throughout the study. SGA infants showed slightly higher energy and protein intakes, even though the energy and protein intakes were not significantly different between the groups of infants at each study point. On the contrary, the accumulation and/or the aberrant distribution of fat mass reported in children and adults born SGA may reflect the long-term fetal programming of adipose tissue alterations, both in terms of quantity and functions [35], and may contribute to the increased risk of developing the metabolic syndrome later in life [36].

The dynamic changes in adiposity that occur during postnatal catch up growth seem to play a critical role in the development of metabolic complications [36]. However, the exact timing of these changes that contributes to the increased later disease risk is still under debate. Ezzahir et al. [34] suggested that the effect of catch-up in body mass index on adiposity in adulthood is mostly detrimental in children born SGA when occurring after 1 y of age.

The strength of the present study relies on the fact that it is a longitudinal study conducted in a relatively large cohort of preterm infants, make observing the changes in growth and body composition more accurate. However, as we aimed to investigate the growth and FM gain according to intrauterine growth pattern and according to postnatal growth, data was analyzed at each study point between groups.

The limitation of the study is that the length of the follow- up was relatively short.

The present study provides preliminary evidence on growth and weight gain composition of preterm infants according to intrauterine growth pattern and according to postnatal growth. Our data suggests that fetal growth pattern influences the potential to rapidly correct anthropometry whereas the restoration of fat stores takes place irrespective of birth weight. Long term follow- up studies are needed to elucidate the metabolic consequences of these findings.
